# (*E*)-4-(β-d-Allopyran­os­yloxy)cinnamyl 4-bromo­phenyl ketone ethanol solvate

**DOI:** 10.1107/S1600536809029687

**Published:** 2009-07-31

**Authors:** Xiu-Juan Yin, Xue Bai, Lei Zheng, Ying Li, Shu-Fan Yin

**Affiliations:** a College of Chemistry, Sichuan University, Chengdu 610064, People’s Republic of China

## Abstract

The title compound, C_21_H_21_BrO_7_·C_2_H_6_O, was synthesized by the Claisen–Schimidt reaction of helicid (systematic name: 4-formyl­phenyl-β-d-allopyran­oside) with 4-bromo­aceto­phenone in ethanol. The pyran ring adopts a chair conformation. In the crystal structure, mol­ecules are linked into a three-dimensional network by inter­molecular O—H⋯O hydrogen bonds.

## Related literature

For helicid and its biological activity, see: Chen *et al.* (1981 ▶); Sha & Mao (1987 ▶). For the synthesis and structure of related compound, see: Fan *et al.* (2007 ▶); Fu *et al.* (2009 ▶); Lv *et al.* (2009 ▶); Yang *et al.* (2009 ▶); Ye *et al.* (2009 ▶).
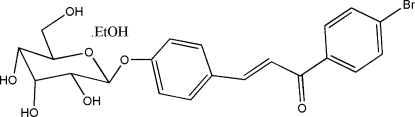


## Experimental

### 

#### Crystal data

C_21_H_21_BrO_7_·C_2_H_6_O
*M* *_r_* = 511.36
Monoclinic, 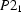

*a* = 10.977 (2) Å
*b* = 7.6518 (15) Å
*c* = 13.259 (3) Å
β = 92.08 (3)°
*V* = 1113.0 (4) Å^3^
*Z* = 2
Mo *K*α radiation
μ = 1.89 mm^−1^
*T* = 113 K
0.20 × 0.16 × 0.12 mm

#### Data collection

Rigaku Saturn CCD area-detector diffractometer
Absorption correction: multi-scan (*CrystalClear*; Rigaku/MSC, 2005 ▶) *T*_min_ = 0.703, *T*_max_ = 0.805
9199 measured reflections
5171 independent reflections
3636 reflections with *I* > 2σ(*I*)
*R*_int_ = 0.039

#### Refinement

*R*[*F* ^2^ > 2σ(*F* ^2^)] = 0.032
*wR*(*F* ^2^) = 0.056
*S* = 0.75
5171 reflections
296 parameters
1 restraint
H-atom parameters constrained
Δρ_max_ = 0.67 e Å^−3^
Δρ_min_ = −0.39 e Å^−3^
Absolute structure: Flack (1983 ▶), 2358 Friedel pairs
Flack parameter: 0.027 (6)

### 

Data collection: *CrystalClear* (Rigaku/MSC, 2005 ▶); cell refinement: *CrystalClear*; data reduction: *CrystalClear*; program(s) used to solve structure: *SHELXS97* (Sheldrick, 2008 ▶); program(s) used to refine structure: *SHELXL97* (Sheldrick, 2008 ▶); molecular graphics: *SHELXTL* (Sheldrick, 2008 ▶); software used to prepare material for publication: *SHELXTL*.

## Supplementary Material

Crystal structure: contains datablocks global, I. DOI: 10.1107/S1600536809029687/rz2355sup1.cif

Structure factors: contains datablocks I. DOI: 10.1107/S1600536809029687/rz2355Isup2.hkl

Additional supplementary materials:  crystallographic information; 3D view; checkCIF report

## Figures and Tables

**Table 1:** Hydrogen-bond geometry (Å, °)

*D*—H⋯*A*	*D*—H	H⋯*A*	*D*⋯*A*	*D*—H⋯*A*
O2—H2⋯O3^i^	0.84	1.97	2.783 (3)	164
O3—H3⋯O7^ii^	0.84	2.05	2.702 (3)	134
O3—H3⋯O4	0.84	2.38	2.786 (3)	110
O4—H4⋯O2^iii^	0.84	1.85	2.677 (3)	166
O5—H5⋯O8^iv^	0.84	1.91	2.678 (3)	152
O8—H8*A*⋯O1^iv^	0.84	2.08	2.893 (3)	163

Symmetry codes: (i) 
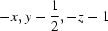
; (ii) 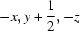; (iii) 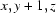; (iv) 
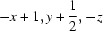
.

**Fig. 1.**
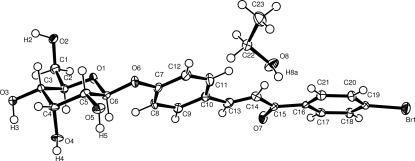
The molecular structure of the title compound, with displacement ellipsoids drawn at the 30% probability level.

## supplementary crystallographic information

### Comment

Helicid (systematic name: 4-formylphenyl-β-D-allopyranoside; Chen *et al.* 1981), is a pure natural compound extracted from the fruit of Helicia Nilagirica Beed, which has been successfully used in the treatment of insomnia in China. Some helicid derivatives have been reported to possess good biological activities (Sha & Mao, 1987). The synthesis and structure of some helicid derivatives heve been recently reported by our group (Fu *et al.* 2009; Lv *et al.* 2009; Yang *et al.* 2009; Ye *et al.* 2009). As a continuation of our studies in this area, we report herein the crystal structure of the title compound.

In the molecule of the title compound (Fig. 1), the average of C–C, C(*sp*^3^)–O and C(*sp*^2^)–O bond lengths in the pyranoside unit are 1.524 (4), 1.421 (4) and 1.241 (3) Å, respectively. The pyran ring adopts chair conformation with the hydroxy group at C4 in axial position and the other substituents at C2, C3 and C5 in equatorial positions. The O1–C2–C3–O3 and O2–C1–C2–O1 torsion angles are -176.5 (2) ° and -59.0 (3) °, respectively, while the O5–C5–C6–O1 and O7–C15–C16–C21 torsion angles are -173.9 (2) ° and -172.2 (3) °, respectively, possibly as a consequence of the presence of O—H···O hydrogen bonds. In the crystal packing, the molecules are linked by intermolecular O—H···O hydrogen bonds (Table 1) involving the hydroxy groups of the pyranoside unit and the ethanol molecule to form a three-dimensional network.

### Experimental

The synthetic method of the title compound was reported elsewhere (Fan *et al.*, 2007). To a solution of helicid (1.420 g, 5 mmol) in 30 ml of anhydrous ethanol, a 10% NaOH aqueous solution were added under ice bath, then 4-bromoacetophenone (1.104 g, 5.5 mmol) was added. The mixture was neutralized with diluted hydrochloric acid, concentrated to half of the original volume, and the resulting precipitate filtered. Colourless single crystals suitable for X-ray analysis were obtained by slow evaporation of an ethanol solution (yield 65%, m.p. 98–100 K).

### Refinement

H atoms were positioned geometrically and refined using a riding model, with C—H = 0.95–0.99 Å, O—H = 0.84 Å, and with *U*_iso_(H) = 1.2*U*_eq_(C) or 1.5*U*_eq_ (C, O) for methyl and hydroxy H atoms.

### Crystal data

**Table:**

C_21_H_21_BrO_7_·C_2_H_6_O	*F*(000) = 528
*M**_r_* = 511.36	*D*_x_ = 1.526 Mg m^−^^3^
Monoclinic, *P*2_1_	Mo *K*α radiation, λ = 0.71073 Å
Hall symbol: P 2yb	Cell parameters from 3521 reflections
*a* = 10.977 (2) Å	θ = 1.5–27.9°
*b* = 7.6518 (15) Å	µ = 1.89 mm^−^^1^
*c* = 13.259 (3) Å	*T* = 113 K
β = 92.08 (3)°	Block, colourless
*V* = 1113.0 (4) Å^3^	0.20 × 0.16 × 0.12 mm
*Z* = 2	

### Data collection

**Table:**

Rigaku Saturn CCD area-detector diffractometer	5171 independent reflections
Radiation source: rotating anode	3636 reflections with *I* > 2σ(*I*)
confocal	*R*_int_ = 0.039
Detector resolution: 7.31 pixels mm^-1^	θ_max_ = 27.9°, θ_min_ = 1.5°
ω and φ scans	*h* = −14→14
Absorption correction: multi-scan (*CrystalClear*; Rigaku/MSC, 2005)	*k* = −10→10
*T*_min_ = 0.703, *T*_max_ = 0.805	*l* = −13→17
9199 measured reflections	

### Refinement

**Table:**

Refinement on *F*^2^	Hydrogen site location: inferred from neighbouring sites
Least-squares matrix: full	H-atom parameters constrained
*R*[*F*^2^ > 2σ(*F*^2^)] = 0.032	*w* = 1/[σ^2^(*F*_o_^2^)] where *P* = (*F*_o_^2^ + 2*F*_c_^2^)/3
*wR*(*F*^2^) = 0.056	(Δ/σ)_max_ = 0.003
*S* = 0.75	Δρ_max_ = 0.67 e Å^−^^3^
5171 reflections	Δρ_min_ = −0.39 e Å^−^^3^
296 parameters	Extinction correction: *SHELXL97* (Sheldrick, 2008)
1 restraint	Extinction coefficient: 0.0242 (11)
Primary atom site location: structure-invariant direct methods	Absolute structure: Flack (1983), 2358 Friedel pairs
Secondary atom site location: difference Fourier map	Flack parameter: 0.027 (6)

### Special details

**Table:**

Geometry. All e.s.d.'s (except the e.s.d. in the dihedral angle between two l.s. planes) are estimated using the full covariance matrix. The cell e.s.d.'s are taken into account individually in the estimation of e.s.d.'s in distances, angles and torsion angles; correlations between e.s.d.'s in cell parameters are only used when they are defined by crystal symmetry. An approximate (isotropic) treatment of cell e.s.d.'s is used for estimating e.s.d.'s involving l.s. planes.
Refinement. Refinement of *F*^2^ against ALL reflections. The weighted *R*-factor *wR* and goodness of fit *S* are based on *F*^2^, conventional *R*-factors *R* are based on *F*, with *F* set to zero for negative *F*^2^. The threshold expression of *F*^2^ > σ(*F*^2^) is used only for calculating *R*-factors(gt) *etc*. and is not relevant to the choice of reflections for refinement. *R*-factors based on *F*^2^ are statistically about twice as large as those based on *F*, and *R*- factors based on ALL data will be even larger.

### Fractional atomic coordinates and isotropic or equivalent isotropic displacement parameters (Å^2^)

**Table:**

	*x*	*y*	*z*	*U*_iso_*/*U*_eq_	
Br1	0.70240 (2)	0.31136 (4)	0.80385 (2)	0.02313 (10)	
O1	0.15927 (15)	0.3593 (2)	−0.22799 (13)	0.0125 (5)	
O2	0.08102 (18)	0.0860 (3)	−0.35522 (15)	0.0166 (5)	
H2	0.0553	0.0678	−0.4147	0.025*	
O3	−0.04022 (16)	0.4996 (3)	−0.44247 (14)	0.0162 (5)	
H3	−0.0788	0.5823	−0.4169	0.024*	
O4	0.05197 (19)	0.7491 (2)	−0.30705 (18)	0.0153 (5)	
H4	0.0691	0.8554	−0.3136	0.023*	
O5	0.32128 (15)	0.7681 (2)	−0.28189 (15)	0.0164 (5)	
H5	0.2777	0.8430	−0.2541	0.025*	
O6	0.32452 (16)	0.4805 (3)	−0.14705 (14)	0.0143 (5)	
O7	0.23659 (17)	0.2120 (3)	0.45816 (14)	0.0192 (5)	
C1	−0.0025 (3)	0.1941 (4)	−0.3055 (2)	0.0168 (8)	
H1A	−0.0209	0.1412	−0.2396	0.020*	
H1B	−0.0796	0.2006	−0.3464	0.020*	
C2	0.0466 (2)	0.3767 (4)	−0.2884 (2)	0.0122 (7)	
H2A	−0.0134	0.4455	−0.2496	0.015*	
C3	0.0726 (3)	0.4741 (4)	−0.3866 (2)	0.0108 (7)	
H3A	0.1279	0.4012	−0.4276	0.013*	
C4	0.1343 (3)	0.6463 (4)	−0.3622 (2)	0.0142 (7)	
H4A	0.1532	0.7077	−0.4264	0.017*	
C5	0.2521 (2)	0.6144 (4)	−0.3006 (2)	0.0127 (7)	
H5A	0.3033	0.5322	−0.3396	0.015*	
C6	0.2173 (2)	0.5219 (4)	−0.2039 (2)	0.0126 (7)	
H6	0.1627	0.5976	−0.1639	0.015*	
C7	0.3110 (3)	0.4342 (4)	−0.0469 (2)	0.0135 (7)	
C8	0.1998 (2)	0.4166 (4)	−0.0018 (2)	0.0145 (7)	
H8	0.1258	0.4363	−0.0394	0.017*	
C9	0.1983 (2)	0.3697 (4)	0.0992 (2)	0.0156 (7)	
H9	0.1221	0.3584	0.1303	0.019*	
C10	0.3045 (2)	0.3390 (4)	0.1562 (2)	0.0136 (7)	
C11	0.4158 (2)	0.3544 (4)	0.1087 (2)	0.0190 (8)	
H11	0.4899	0.3316	0.1457	0.023*	
C12	0.4188 (2)	0.4030 (4)	0.0076 (2)	0.0178 (7)	
H12	0.4947	0.4147	−0.0240	0.021*	
C13	0.2936 (2)	0.2962 (5)	0.26334 (19)	0.0167 (7)	
H13	0.2127	0.2824	0.2851	0.020*	
C14	0.3805 (2)	0.2741 (4)	0.3339 (2)	0.0141 (7)	
H14	0.4640	0.2796	0.3177	0.017*	
C15	0.3446 (3)	0.2406 (4)	0.4391 (2)	0.0142 (7)	
C16	0.4376 (2)	0.2468 (4)	0.5234 (2)	0.0116 (7)	
C17	0.4006 (3)	0.2023 (4)	0.6211 (2)	0.0146 (7)	
H17	0.3199	0.1618	0.6302	0.017*	
C18	0.4800 (2)	0.2172 (4)	0.7029 (2)	0.0152 (7)	
H18	0.4547	0.1872	0.7684	0.018*	
C19	0.5973 (2)	0.2762 (4)	0.6893 (2)	0.0150 (8)	
C20	0.6379 (2)	0.3148 (5)	0.59468 (18)	0.0139 (6)	
H20	0.7194	0.3525	0.5864	0.017*	
C21	0.5579 (2)	0.2977 (5)	0.51141 (18)	0.0137 (6)	
H21	0.5857	0.3211	0.4457	0.016*	
O8	0.7446 (2)	0.5264 (3)	0.15901 (15)	0.0236 (5)	
H8A	0.7702	0.6293	0.1661	0.035*	
C22	0.7527 (2)	0.4749 (4)	0.0559 (2)	0.0186 (7)	
H22A	0.7139	0.5652	0.0120	0.022*	
H22B	0.7076	0.3642	0.0448	0.022*	
C23	0.8844 (2)	0.4500 (4)	0.0265 (2)	0.0283 (9)	
H23A	0.9281	0.5612	0.0334	0.042*	
H23B	0.8862	0.4100	−0.0437	0.042*	
H23C	0.9237	0.3626	0.0708	0.042*	

### Atomic displacement parameters (Å^2^)

**Table:**

	*U*^11^	*U*^22^	*U*^33^	*U*^12^	*U*^13^	*U*^23^
Br1	0.02318 (17)	0.03083 (19)	0.01506 (14)	−0.0005 (2)	−0.00380 (11)	−0.0020 (2)
O1	0.0135 (10)	0.0100 (12)	0.0137 (10)	−0.0031 (9)	−0.0042 (8)	0.0028 (8)
O2	0.0266 (13)	0.0125 (13)	0.0105 (12)	0.0026 (11)	−0.0005 (10)	−0.0007 (10)
O3	0.0193 (12)	0.0162 (12)	0.0128 (12)	0.0055 (10)	−0.0041 (9)	−0.0025 (10)
O4	0.0169 (11)	0.0075 (11)	0.0216 (12)	0.0025 (9)	0.0040 (9)	−0.0012 (9)
O5	0.0157 (11)	0.0123 (14)	0.0214 (12)	−0.0043 (9)	0.0046 (9)	−0.0034 (9)
O6	0.0117 (11)	0.0202 (13)	0.0108 (11)	−0.0012 (9)	−0.0022 (9)	0.0021 (10)
O7	0.0108 (11)	0.0321 (14)	0.0148 (12)	−0.0053 (10)	0.0011 (9)	−0.0017 (10)
C1	0.020 (2)	0.0157 (18)	0.0144 (17)	0.0003 (15)	0.0014 (15)	0.0018 (15)
C2	0.0133 (17)	0.0116 (16)	0.0118 (16)	−0.0002 (13)	−0.0007 (13)	0.0000 (13)
C3	0.0106 (16)	0.0127 (17)	0.0091 (17)	0.0015 (14)	−0.0009 (13)	0.0010 (14)
C4	0.0166 (18)	0.0129 (17)	0.0135 (17)	0.0049 (14)	0.0071 (14)	0.0010 (14)
C5	0.0142 (16)	0.0104 (17)	0.0135 (16)	−0.0022 (13)	0.0021 (13)	−0.0012 (13)
C6	0.0142 (17)	0.0122 (16)	0.0113 (16)	0.0019 (14)	−0.0012 (13)	−0.0049 (14)
C7	0.0124 (16)	0.0186 (18)	0.0096 (16)	−0.0005 (14)	0.0014 (13)	−0.0024 (14)
C8	0.0102 (16)	0.0181 (18)	0.0148 (17)	0.0019 (14)	−0.0060 (13)	−0.0002 (14)
C9	0.0111 (15)	0.0218 (18)	0.0139 (16)	0.0004 (13)	0.0028 (12)	−0.0018 (13)
C10	0.0123 (14)	0.014 (2)	0.0145 (14)	−0.0020 (15)	−0.0004 (11)	−0.0002 (14)
C11	0.0130 (15)	0.029 (2)	0.0148 (15)	−0.0016 (14)	−0.0026 (12)	0.0016 (14)
C12	0.0090 (16)	0.0261 (19)	0.0185 (17)	−0.0015 (14)	0.0011 (13)	−0.0065 (14)
C13	0.0176 (15)	0.0177 (17)	0.0152 (14)	−0.0017 (19)	0.0037 (11)	−0.0018 (18)
C14	0.0130 (15)	0.014 (2)	0.0159 (15)	−0.0009 (14)	0.0032 (12)	0.0025 (14)
C15	0.0182 (17)	0.0133 (16)	0.0111 (16)	0.0031 (13)	−0.0010 (13)	−0.0029 (13)
C16	0.0161 (16)	0.0104 (15)	0.0084 (15)	0.0018 (13)	0.0003 (12)	−0.0021 (12)
C17	0.0119 (16)	0.0166 (18)	0.0155 (17)	0.0010 (13)	0.0045 (13)	0.0006 (14)
C18	0.0198 (18)	0.0188 (18)	0.0072 (15)	0.0014 (14)	0.0009 (13)	0.0029 (13)
C19	0.0176 (15)	0.013 (2)	0.0136 (14)	0.0049 (14)	−0.0041 (12)	−0.0014 (14)
C20	0.0106 (13)	0.0156 (14)	0.0156 (13)	−0.004 (2)	−0.0002 (10)	0.002 (2)
C21	0.0144 (14)	0.0167 (16)	0.0103 (13)	−0.0015 (19)	0.0045 (10)	0.0022 (18)
O8	0.0384 (14)	0.0154 (13)	0.0172 (13)	−0.0044 (11)	0.0052 (11)	−0.0017 (11)
C22	0.0192 (18)	0.0199 (19)	0.0164 (18)	0.0015 (15)	−0.0031 (14)	−0.0034 (15)
C23	0.0161 (18)	0.037 (2)	0.031 (2)	0.0019 (16)	−0.0027 (15)	−0.0014 (18)

### Geometric parameters (Å, °)

**Table:**

Br1—C19	1.893 (3)	C9—C10	1.385 (3)
O1—C6	1.429 (3)	C9—H9	0.9500
O1—C2	1.456 (3)	C10—C11	1.400 (3)
O2—C1	1.415 (3)	C10—C13	1.468 (3)
O2—H2	0.8400	C11—C12	1.394 (4)
O3—C3	1.433 (3)	C11—H11	0.9500
O3—H3	0.8400	C12—H12	0.9500
O4—C4	1.421 (3)	C13—C14	1.323 (3)
O4—H4	0.8400	C13—H13	0.9500
O5—C5	1.417 (3)	C14—C15	1.485 (4)
O5—H5	0.8400	C14—H14	0.9500
O6—C7	1.387 (3)	C15—C16	1.487 (3)
O6—C6	1.411 (3)	C16—C21	1.391 (3)
O7—C15	1.241 (3)	C16—C17	1.413 (4)
C1—C2	1.511 (4)	C17—C18	1.371 (4)
C1—H1A	0.9900	C17—H17	0.9500
C1—H1B	0.9900	C18—C19	1.382 (3)
C2—C3	1.535 (4)	C18—H18	0.9500
C2—H2A	1.0000	C19—C20	1.378 (3)
C3—C4	1.511 (4)	C20—C21	1.392 (3)
C3—H3A	1.0000	C20—H20	0.9500
C4—C5	1.524 (4)	C21—H21	0.9500
C4—H4A	1.0000	O8—C22	1.429 (3)
C5—C6	1.526 (3)	O8—H8A	0.8400
C5—H5A	1.0000	C22—C23	1.523 (4)
C6—H6	1.0000	C22—H22A	0.9900
C7—C12	1.384 (4)	C22—H22B	0.9900
C7—C8	1.385 (4)	C23—H23A	0.9800
C8—C9	1.387 (4)	C23—H23B	0.9800
C8—H8	0.9500	C23—H23C	0.9800
			
C6—O1—C2	114.0 (2)	C8—C9—H9	119.0
C1—O2—H2	109.5	C9—C10—C11	118.2 (3)
C3—O3—H3	109.5	C9—C10—C13	118.0 (2)
C4—O4—H4	109.5	C11—C10—C13	123.8 (2)
C5—O5—H5	109.5	C12—C11—C10	120.4 (3)
C7—O6—C6	116.9 (2)	C12—C11—H11	119.8
O2—C1—C2	112.1 (2)	C10—C11—H11	119.8
O2—C1—H1A	109.2	C7—C12—C11	120.0 (3)
C2—C1—H1A	109.2	C7—C12—H12	120.0
O2—C1—H1B	109.2	C11—C12—H12	120.0
C2—C1—H1B	109.2	C14—C13—C10	129.2 (2)
H1A—C1—H1B	107.9	C14—C13—H13	115.4
O1—C2—C1	106.8 (2)	C10—C13—H13	115.4
O1—C2—C3	109.3 (2)	C13—C14—C15	118.5 (2)
C1—C2—C3	113.5 (3)	C13—C14—H14	120.8
O1—C2—H2A	109.0	C15—C14—H14	120.8
C1—C2—H2A	109.0	O7—C15—C14	120.6 (2)
C3—C2—H2A	109.0	O7—C15—C16	119.2 (3)
O3—C3—C4	111.4 (2)	C14—C15—C16	120.2 (2)
O3—C3—C2	108.7 (2)	C21—C16—C17	118.6 (2)
C4—C3—C2	109.8 (2)	C21—C16—C15	123.4 (2)
O3—C3—H3A	109.0	C17—C16—C15	118.0 (2)
C4—C3—H3A	109.0	C18—C17—C16	120.6 (3)
C2—C3—H3A	109.0	C18—C17—H17	119.7
O4—C4—C3	107.7 (2)	C16—C17—H17	119.7
O4—C4—C5	110.8 (2)	C17—C18—C19	119.5 (3)
C3—C4—C5	109.9 (2)	C17—C18—H18	120.2
O4—C4—H4A	109.5	C19—C18—H18	120.2
C3—C4—H4A	109.5	C20—C19—C18	121.5 (2)
C5—C4—H4A	109.5	C20—C19—Br1	119.5 (2)
O5—C5—C4	113.6 (2)	C18—C19—Br1	119.0 (2)
O5—C5—C6	112.8 (2)	C19—C20—C21	119.1 (2)
C4—C5—C6	106.9 (2)	C19—C20—H20	120.4
O5—C5—H5A	107.8	C21—C20—H20	120.4
C4—C5—H5A	107.8	C16—C21—C20	120.6 (2)
C6—C5—H5A	107.8	C16—C21—H21	119.7
O6—C6—O1	106.3 (2)	C20—C21—H21	119.7
O6—C6—C5	108.9 (2)	C22—O8—H8A	109.5
O1—C6—C5	109.8 (2)	O8—C22—C23	111.9 (2)
O6—C6—H6	110.6	O8—C22—H22A	109.2
O1—C6—H6	110.6	C23—C22—H22A	109.2
C5—C6—H6	110.6	O8—C22—H22B	109.2
C12—C7—C8	120.5 (3)	C23—C22—H22B	109.2
C12—C7—O6	115.1 (2)	H22A—C22—H22B	107.9
C8—C7—O6	124.4 (3)	C22—C23—H23A	109.5
C7—C8—C9	118.9 (3)	C22—C23—H23B	109.5
C7—C8—H8	120.5	H23A—C23—H23B	109.5
C9—C8—H8	120.5	C22—C23—H23C	109.5
C10—C9—C8	122.1 (3)	H23A—C23—H23C	109.5
C10—C9—H9	119.0	H23B—C23—H23C	109.5
			
C6—O1—C2—C1	−179.3 (2)	C7—C8—C9—C10	−0.4 (5)
C6—O1—C2—C3	57.5 (3)	C8—C9—C10—C11	−0.7 (5)
O2—C1—C2—O1	−59.0 (3)	C8—C9—C10—C13	177.9 (3)
O2—C1—C2—C3	61.6 (3)	C9—C10—C11—C12	1.3 (5)
O1—C2—C3—O3	−176.5 (2)	C13—C10—C11—C12	−177.2 (3)
C1—C2—C3—O3	64.4 (3)	C8—C7—C12—C11	−0.3 (4)
O1—C2—C3—C4	−54.5 (3)	O6—C7—C12—C11	−179.5 (3)
C1—C2—C3—C4	−173.6 (2)	C10—C11—C12—C7	−0.8 (5)
O3—C3—C4—O4	57.7 (3)	C9—C10—C13—C14	−174.7 (4)
C2—C3—C4—O4	−62.7 (3)	C11—C10—C13—C14	3.8 (6)
O3—C3—C4—C5	178.6 (2)	C10—C13—C14—C15	176.7 (3)
C2—C3—C4—C5	58.1 (3)	C13—C14—C15—O7	9.0 (4)
O4—C4—C5—O5	−66.2 (3)	C13—C14—C15—C16	−169.0 (3)
C3—C4—C5—O5	174.9 (2)	O7—C15—C16—C21	−172.2 (3)
O4—C4—C5—C6	58.8 (3)	C14—C15—C16—C21	5.7 (4)
C3—C4—C5—C6	−60.1 (3)	O7—C15—C16—C17	6.2 (4)
C7—O6—C6—O1	−76.7 (3)	C14—C15—C16—C17	−175.8 (3)
C7—O6—C6—C5	165.1 (2)	C21—C16—C17—C18	3.1 (4)
C2—O1—C6—O6	−179.19 (19)	C15—C16—C17—C18	−175.5 (3)
C2—O1—C6—C5	−61.5 (3)	C16—C17—C18—C19	0.0 (4)
O5—C5—C6—O6	−57.9 (3)	C17—C18—C19—C20	−2.3 (5)
C4—C5—C6—O6	176.6 (2)	C17—C18—C19—Br1	175.7 (2)
O5—C5—C6—O1	−173.9 (2)	C18—C19—C20—C21	1.5 (5)
C4—C5—C6—O1	60.6 (3)	Br1—C19—C20—C21	−176.5 (3)
C6—O6—C7—C12	−176.9 (3)	C17—C16—C21—C20	−3.9 (5)
C6—O6—C7—C8	3.9 (4)	C15—C16—C21—C20	174.6 (3)
C12—C7—C8—C9	0.9 (4)	C19—C20—C21—C16	1.6 (6)
O6—C7—C8—C9	−179.9 (3)		

### Hydrogen-bond geometry (Å, °)

**Table:**

*D*—H···*A*	*D*—H	H···*A*	*D*···*A*	*D*—H···*A*
O2—H2···O3^i^	0.84	1.97	2.783 (3)	164
O3—H3···O7^ii^	0.84	2.05	2.702 (3)	134
O3—H3···O4	0.84	2.38	2.786 (3)	110
O4—H4···O2^iii^	0.84	1.85	2.677 (3)	166
O5—H5···O8^iv^	0.84	1.91	2.678 (3)	152
O8—H8A···O1^iv^	0.84	2.08	2.893 (3)	163

Symmetry codes: (i) −*x*, *y*−1/2, −*z*−1; (ii) −*x*, *y*+1/2, −*z*; (iii) *x*, *y*+1, *z*; (iv) −*x*+1, *y*+1/2, −*z*.

## References

[bb1] Chen, W. S., Lu, S. D. & Eberhard, B. (1981). *Liebigs Ann. Chem.* **10**, 1893–1895.

[bb2] Fan, B., Li, J. L., Li, Y. & Yin, S. F. (2007). *Chin. J. Org. Chem.* **27**, 1150–1154.

[bb3] Flack, H. D. (1983). *Acta Cryst.* A**39**, 876–881.

[bb4] Fu, L., Yin, X., Zheng, L., Li, Y. & Yin, S. (2009). *Acta Cryst.* E**65**, o679. 10.1107/S1600536809007260 PMC2968846 21582423

[bb5] Lv, S.-M., Zheng, L., Zhao, H., Li, Y. & Yin, S.-F. (2009). *Acta Cryst.* E**65**, o290. 10.1107/S1600536809000944 PMC2968321 21581901

[bb6] Rigaku/MSC (2005). *CrystalClear* and *CrystalStructure* Rigaku/MSC Inc., The Woodlands, Texas, USA.

[bb7] Sha, J. Z. & Mao, H. K. (1987). *Chin. Pharm. Bull.* **22**, 27–30.

[bb8] Sheldrick, G. M. (2008). *Acta Cryst.* A**64**, 112–122. 10.1107/S0108767307043930 18156677

[bb9] Yang, C., Luo, H., Yin, X., Li, Y. & Yin, S. (2009). *Acta Cryst.* E**65**, o634. 10.1107/S1600536809006424 PMC2968659 21582283

[bb10] Ye, D., Zhang, K., Chen, H., Yin, S. & Li, Y. (2009). *Acta Cryst.* E**65**, o1338. 10.1107/S1600536809018248 PMC2969774 21583191

